# Infant and young child feeding practices and nutritional status in Bhutan*

**DOI:** 10.1111/mcn.12762

**Published:** 2018-11-29

**Authors:** Rebecca K. Campbell, Víctor M. Aguayo, Yunhee Kang, Laigden Dzed, Vandana Joshi, Jillian Waid, Suvadra Datta Gupta, Nancy Haselow, Keith P. West,

**Affiliations:** ^1^ Center for Human Nutrition Johns Hopkins Bloomberg School of Public Health Baltimore Maryland USA; ^2^ UNICEF New York New York USA; ^3^ Ministry of Health Government of Bhutan Thimphu Bhutan; ^4^ UNICEF Bhutan Thimphu Bhutan; ^5^ HKI Dhaka Bangladesh; ^6^ HKI Regional Office, Asia Pacific Phnom Penh Cambodia

**Keywords:** breastfeeding, complementary feeding, diet quality, overweight, South Asia, undernutrition

## Abstract

In South Asia, childhood undernutrition persists while overweight is increasing. Internationally recommended infant and young child feeding (IYCF) practices promote healthy nutritional status; however, little is known about IYCF in Bhutan, investigated here using 2015 National Nutrition Survey data. WHO/UNICEF IYCF indicators, anthropometry and household socio‐economic status were available for 441 children <24 months. Stunting, wasting, and underweight prevalence (<−2Z length‐for‐age [LAZ], weight‐for‐age, [WAZ] and weight‐for‐length [WLZ], respectively) were 15%, 9%, and 5%, respectively, whereas overweight (WLZ >2) prevalence was 6%. In survey‐design‐adjusted analyses, 52% of mothers of 0‐ to 5‐month olds reported exclusive breastfeeding (EBF), with EBF less common for girls than boys (OR: 0.2 [95% CI: 0.1–0.9]). Although 61% of children were breastfed at 2 years and 75% of children >6 months met a minimum daily meal frequency, only 18% of children 6–23 months met minimum dietary diversity. IYCF was unassociated with risk of stunting, wasting, or underweight, possibly due to relatively low prevalence of anthropometric failure and small sample size. However, currently‐breastfed children were less often overweight [OR: ~0.1 (95% upper limit ≤1.0)]. Neither breastfeeding nor most complementary feeding practices differed by socio‐economic status, but children in the highest two fifth of a wealth index had 7.8 (1.3–46.9) and 5.3 (1.1–25.2) times greater odds than children in the lowest fifth of meeting minimum dietary diversity criteria. Low rates of EBF, given possible protection of breastfeeding against overweight, and inadequate dietary diversity offer evidence to guide future program interventions to improve nutritional status of young children.

**Key messages**

- Although some infant and young child feeding practices in Bhutan align with World Health Organization guidelines, others, especially exclusive breastfeeding and dietary diversity, remain inadequate.
- Adherence to recommended breastfeeding practices is associated with a lower risk of infants and young children being overweight or obese.
- Breastfeeding patterns appear not to be constrained by maternal and household factors, implying breastfeeding promotion can succeed across all socio‐economic strata of Bhutanese society.
- Dietary diversity may be limited by household socio‐economic means, suggesting a need to reduce barriers to accessing a diverse, nutritious diet for children from more impoverished households.

## 1 INTRODUCTION

South Asia faces the highest rates and greatest burden of undernutrition among children and women worldwide (Black et al., 2013). In recent years, overweight and obesity have also increased despite persistent undernutrition, creating a so‐called “double‐burden” of malnutrition (Haddad, Cameron, & Barnett, 2015). The concurrence of undernutrition and overweight in the same communities and households has prompted research into potential shared causes and consideration of intervention strategies addressing both issues (Popkin, 2001; Popkin, Adair, & Ng, 2012).

Appropriate breastfeeding and complementary feeding practices in the first 2 years of life are conduits for good nutrition, offering protection against both undernutrition and overweight in the short and longer terms. International recommendations for breastfeeding and complementary feeding, known collectively as infant and young child feeding (IYCF), were operationalized by WHO/UNICEF for use in nutrition surveys (Daelmans, Dewey, Arimond, & Working Group on Infant Young Child Feeding Indicators, 2009). In studies utilizing these indicators, recommended IYCF practices were found to be protective against undernutrition in some, but not all, study settings (Aguayo, Nair, Badgaiyan, & Krishna, 2016; Lamichhane et al., 2016; Menon, Bamezai, Subandoro, Ayoya, & Aguayo, 2015; Zongrone, Winskell, & Menon, 2012), whereas associations with overweight are not well documented.

In Bhutan, nutrition and health surveys conducted over the past two decades suggest a landscape of mild to moderate undernutrition with rapid but somewhat uneven improvements (Aguayo, Badgaiyan, & Paintal, 2015). In 2015, a new national survey of women and children's nutrition, called the National Nutrition Survey (NNS) Bhutan 2015, was conducted by the Ministry of Health in conjunction with the National Statistical Bureau, Khesar Gyalpo University of Medical Sciences of Bhutan and UNICEF with technical support from Helen Keller International. Initial reported findings reveal continued improvement with pockets of lingering undernutrition (Nutrition Program, Department of Public Health, & Ministry of Health, 2015). Stunting, for example, was observed in 21% of children <5 years of age, whereas underweight and wasting were much lower, and regional and urban–rural differences were apparent. Little is known about whether IYCF practices differ by context in their associations with child nutritional status. Therefore, our analysis aims to investigate IYCF practices and their associations with the nutritional status of infants and young children (<24 months) in Bhutan.

## 2 METHODS

### 2.1 Study design

Data for this study come from the NNS Bhutan 2015. The survey aimed to describe the nutrition situation of children under five, adolescent girls, and women of reproductive age. A multilevel sampling strategy was used to generate estimates representative at the national level and for the Western, Central, and Eastern regions and urban and rural areas of the country. The full details of the survey implementation including the sampling strategy are described in the survey report (Nutrition Program, Department of Public Health, & Ministry of Health, 2015). Briefly, two districts (Dzongkhags) were randomly selected from each region (West, Central, East), within which smaller administrative units (Chiwogs or enumeration areas) were randomly selected in proportion to the urban–rural make‐up in each region. Within each selected Chiwog and enumeration area, 12 households were systematically selected following a random start. An additional purposively sampled district was surveyed but not included in this analysis.

In each selected household, interviewers administered a questionnaire about household members, durable and productive asset ownership, and the physical structure of the family's home, including access to improved water and sanitation facilities. A second questionnaire asking about pregnancy care and IYCF was administered only to the mother of the youngest child under age 2 years in the household. Length or height and weight were measured for all children under 5 years living in selected households.

### 2.2 Data management

All children less than 24 months of age for whom the feeding questionnaire was completed were included in the present analysis. IYCF indicators were defined according to the WHO/UNICEF guidelines to the extent possible (World Health Organization, 2010). Detailed definitions and equations, along with all survey‐specific modifications, are listed in Table S1. The wording of some NNS 2015 questions differed from the recommended wording, generally with greater detail in the NNS. Two indicators, intake of iron‐rich foods and median duration of breastfeeding, could not be calculated with the available information. Additionally, frequency of feeding milk was not queried as it is not considered a culturally relevant practice (V. Joshi, personal communication, 2017), so minimum meal frequency and minimum acceptable diet were calculated assuming no animal milk was consumed by non‐breastfed children. Two indicators beyond those described by WHO were created for this survey to utilize data collected on colostrum and prelacteal feeding at the time of birth; both were dichotomized as yes or no.

Reported food consumption in the past 24 hr was categorized into seven food groups according to the IYCF indicator recommendations: grains/staples, legumes, dairy, flesh foods, eggs, vitamin A‐rich fruits and vegetables, and other fruits and vegetables. The minimum dietary diversity (MDD) indicator was calculated based on these food groups. Additionally, mean weekly intakes were calculated by multiplying the mean of each food group's reported 24‐hr intake by seven to generate values more easily interpreted as representing typical dietary intake.

Household and maternal characteristics were categorized based on examination of their variability and value ranges. A wealth index previously developed by the Bhutan National Statistics Bureau using principal component analysis was used in the NNS 2015 report and retained for this analysis. The primary building material used for the home structure was collapsed to two categories: concrete/cement and mud/wood/other. Improved sanitation was defined according to WHO guidelines, including restricting to facilities not shared with other households (World Health Organization & UNICEF, 2006). Improved water source was defined according to Bhutan's criterion, which is water piped into the household. Household food security was assessed with four questions based on the Household Food Insecurity Access Scale (Coates, Swindale, & Bilinksy, 2007) about the household's experience in the prior month; an affirmative answer to at least one question indicated household food insecurity.

Child length‐for‐age (LAZ), weight‐for‐age (WAZ) and weight‐for‐length (WLZ) Z‐scores were calculated relative to the WHO reference (World Health Organization Multicentre Growth Reference Study Group, 2006). Stunting, underweight, and wasting were defined as LAZ, WAZ, and WLZ, respectively, <−2; overweight/obesity was defined as WLZ >2.

### 2.3 Statistical analysis

Data were first explored for trends, outliers, and non‐normality. Tabulations of household and maternal characteristics were generated at the national level and by region and area (urban/rural), the survey strata. Subsequent analyses used the “svy” commands in Stata to account for the sampling strategy. A detailed treatment of these commands can be found in StataCorp (2015) and O'Donnell, Doorslaer, Wagstaff, and Lidelow (2008), but, briefly, the programme applies survey weights to point estimates and adjusts variance measures for clustering of values within sampling units.

Survey‐adjusted logistic regression models were developed for univariate associations between dichotomous anthropometric outcomes and IYCF indicators. Each IYCF indicator was included as the independent variable in a separate model, as multivariable models were not feasible because of different eligible age ranges for each IYCF indicator. Logistic regression models were also developed for univariate associations between IYCF indicators and maternal and household characteristics. Multivariable models combining household characteristics into a single model for each indicator were explored but not presented due to small sample size within survey strata and, thus, limited power. All analyses were done using Stata, version 14.1 (StataCorp, College Station, TX).

## 3 RESULTS

The NNS Bhutan 2015 included 441 children under age 2 years for whom IYCF and anthropometric data were available. In one case where multiple children in the same household had IYCF data, only the youngest was retained in the data set. Half of assessed children were male and half female. Most households owned land (77%) and livestock (cattle/buffalo/yaks, 92%, and poultry, 79%). A majority also owned a television (66%) and 17% owned a family car. Coverage of improved sanitation facilities and improved water source were 67% and 84%, respectively. Still, approximately 40% of mothers of children under 2 years reported no formal or informal education. National prevalence rates of stunting (15%), wasting (5%), underweight (9%), and overweight (6%) in children under 2 years were moderately low. Only stunting varied by region, affecting 6% of children in the Central region versus 16% and 19% in the West and East regions, respectively. For all indicators, there appeared to be less undernutrition in urban than rural areas, but the differences were small (Table S2).

**Figure 1:**
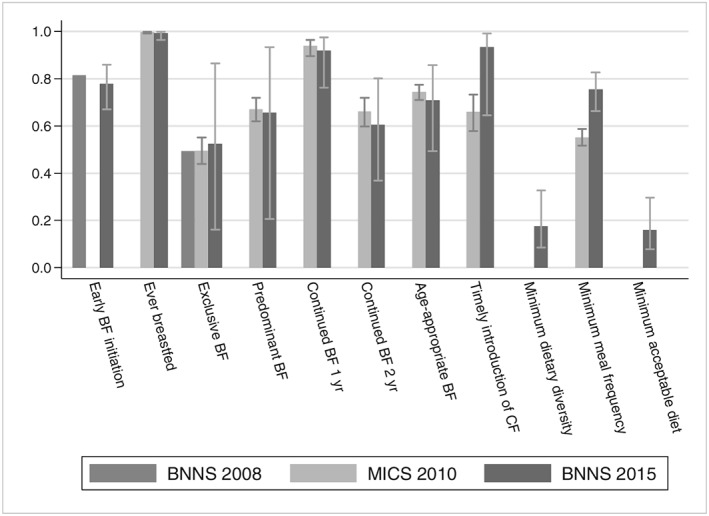
Reported infant and young child feeding practice indicators in Bhutan in the 2008, 2010, and 2015 national nutrition surveys. Specific questionnaire items for infant and young child feeding practices and indicator definitions were not necessarily uniform across surveys, though every attempt was made to present comparable data from the three surveys. In particular, the 2008 NNS preceded the publication of the UNICEF infant and young child feeding assessment guidelines. Abbreviations: BF = breastfeeding; BNNS = Bhutan National Nutrition Survey; CF = complementary feeding; MICS = Multiple Indicator Cluster Survey

**Figure 2:**
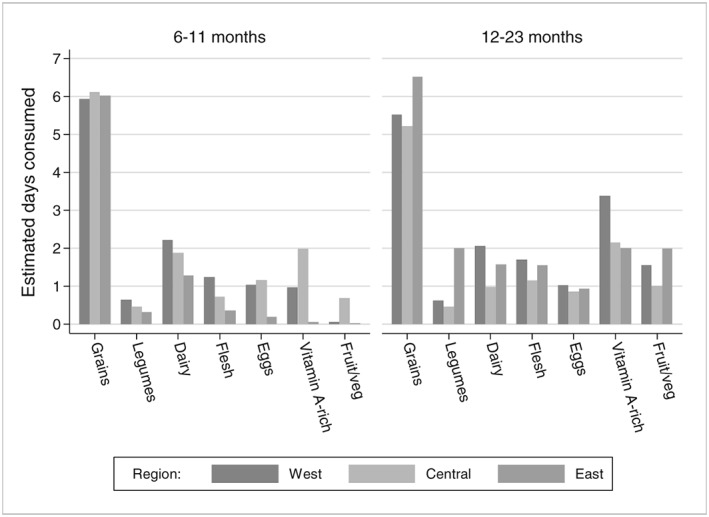
Estimated mean weekly food group consumption by child age and region in the National Nutrition Survey Bhutan 2015. Weekly intakes were estimated from maternal‐reported recall of 24‐hr intakes of a list of foods. Foods were each assigned to one of seven food groups and mean 24‐hr intakes within each age and regional subset of the sample were multiplied by seven to give mean weekly consumption. Regional estimates were generated using “svy, subpop(*region*)” command with region indicator variable specified to account for survey design

Breastfeeding was universal (99% ever breastfed), with 61% of children still breastfeeding through the second year of life (Figure 1). Initiation of breastfeeding within 1 hr after birth was reported by 78% of mothers, with 95% reporting having fed colostrum and only 4% a prelacteal feed. Of mothers nationwide with infants <6 months, 52% reported exclusive breastfeeding. Complementary foods were given to 93% of children 6–8 months of age. Among children 6–23 months of age, 75% and 18% of mothers reported meeting criteria for minimum meal frequency and dietary diversity, respectively, with rates higher in urban than in rural areas for dietary diversity (24% vs. 13%) but lower for meal frequency in urban areas (67%) than in rural (82%; Table S3). We estimated that children consumed grains daily and other food groups up to three times per week, on average (Figure 2). No particular food groups other than grains appeared to dominate children's diets. Frequency of nonstaple food consumption reportedly increased with age, most noticeably for vegetables and fruits and for children living in the East region.

IYCF practices were not associated with the relative odds of being undernourished (Table 1). In contrast, the risk of overweight tended to be lower in children for whom WHO breastfeeding criteria were met. Specifically, exclusive and predominant breastfeeding <6 months and continued breastfeeding at 2 years were associated with reduced odds of overweight (0.1 [0.0–1.0], 0.1 [0.0–0.6], and 0.0 [0.0–0.4], respectively).

**Table 1:** Associations between children's anthropometric measures and mothers' reported infant and young child feeding practices in children <2 years of age in Bhutan

Indicator^a^	Stunting (LAZ < −2)	Wasting (WLZ < −2)	Underweight (WAZ < −2)	Overweight/obesity (WLZ > 2)
	OR (95% CI)^b^	OR (95% CI)	OR (95% CI)	OR (95% CI)
Early initiation of BF	0.6 (0.2, 2.4)	0.6 (0.2, 2.2)	0.7 (0.1, 8.0)	1.1 (0.2, 5.0)
Exclusive BF <6 months	0.8 (0.1, 6.3)	0.4 (0.0, 6.6)	0.4 (0.0, 10.3)	0.1 (0.0, 1.0)*
Predominant BF <6 months	1.9 (0.0, 153.9)	—	1.0 (1.0, 1.0)	0.1 (0.0, 0.6)**
Continued BF, 1 year.	0.7 (0.0, 13.1)	—	1.0 (1.0, 1.0)	—
Continued BF, 2 years.	0.4 (0.1, 2.0)	0.7 (0.0, 11.1)	1.4 (0.2, 11.5)	0.0 (0.0, 0.4)**
Age‐appropriate BF	0.6 (0.2, 1.6)	1.0 (0.2, 5.6)	0.6 (0.2, 1.6)	0.4 (0.1, 1.7)
Fed prelacteal	3.2 (0.8, 13.3)*	1.9 (0.1, 31.9)	1.2 (0.1, 18.3)	—
Fed colostrum	0.5 (0.1, 2.2)	1.2 (0.0, 50.1)	0.4 (0.0, 5.0)	26.0 (1.1, 598.2)**
Timely introduction of CF	1.0 (1.0, 1.0)	—	1.0 (1.0, 1.0)	—
Minimum dietary diversity	0.4 (0.0, 3.2)	0.3 (0.0, 8.1)	0.2 (0.0, 2.2)	0.5 (0.0, 33.5)
Minimum meal frequency	1.7 (0.4, 7.2)	1.2 (0.2, 6.5)	1.8 (0.1, 22.9)	1.3 (0.2, 10.4)
Minimum acceptable diet	0.4 (0.0, 3.9)	0.3 (0.0, 9.7)	0.2 (0.0, 2.7)	0.5 (0.0, 37.7)

*Note*. LAZ = length‐for‐age; WAZ = weight‐for‐age; WLZ = weight‐for‐length; BF = breastfeeding; CF = complementary feeding.

a Full indicator definitions can be found in Table S1.

b Separate models were generated for each infant and young child feeding practice. Stars indicate significant *p* value for the infant and young child feeding coefficient.

*** *p* ≤ .01.

** *p* ≤ .05.

* *p* ≤ .1.

Exclusivity of breastfeeding <6 months and breastfeeding at 2 years of age were not associated with any assessed household or maternal characteristics (Table S4). Girls, however, were less likely to be exclusively breastfed than boys <6 months (OR: 0.2 [95% CI: 0.1–0.9]). Children's complementary feeding diets exhibited a dose‐responsive increased odds of reaching MDD (≥4 groups per day) in households classified between the third and fourth and above the fourth quintile of the wealth index (5.3 [1.1–25.2] and 7.8 [1.3–46.9], respectively) compared to those in households below the first quintile. Children in households with improved versus unimproved sanitation had a 2.6 times (95% CI: 0.7–9.4) greater odds of meeting MDD. Living in a rural versus urban location may have been associated with a reduced odds of reaching the MDD (0.5 [0.2–1.3]).

## 4 DISCUSSION

Based on this 2015 national survey, IYCF practices in Bhutan are suboptimal relative to WHO/UNICEF recommendations: Only 16% of breastfed children 6–23 months old are fed a minimum acceptable diet, whereas 71% of children <24 months are adequately breastfed. Still, we observed a relatively normal anthropometric profile in Bhutan, which may be explained in part by good child feeding practices relative to other South Asian countries.

Repeated surveys in Bhutan have suggested a dynamic IYCF situation in the country with, for example, increases being observed in the timely introduction of complementary foods and meal frequencies from 2010 to 2015 (as seen in Figure 1), that could partly explain an improved nutritional status of children. During this time period, the prevalence of stunting among children <5 years of age decreased by a third, from 33% to 21%, yielding an average reduction of 2.4 percentage points per year (Kang et al., under review, 2017), a decline that exceeds rates recently reported in Bangladesh and Nepal (Headey & Hoddinott, 2014). Attention to early childhood nutrition in the country's 5‐year development plans (Government of Bhutan, 2015), coupled with rapid economic growth (Tobgay, Dophu, Torres, & Na‐Bangchang, 2011) and expansion of health centres to be accessible, within a few hours, of every resident of Bhutan (Tobgay, Dorji, Pelzom, & Gibbons, 2011), may be contributing to observed gains in linear growth.

Notwithstanding a robust reduction in stunting and relatively low risk of wasting malnutrition in the country, some IYCF practices and trends over time suggest a need for improvement. For example, only half of surveyed children under 6 months of age were exclusively breastfed. Differential rates of exclusive breastfeeding by sex, as observed in Bhutan, have not typically been reported from surveys elsewhere in the region (Dibley et al., 2010), although shorter duration of breastfeeding in girls than boys has been described in India (Fledderjohann et al., 2014). Our finding of less exclusive breastfeeding in girls merits further inquiry. Exclusive breastfeeding may protect children from both underweight and overweight, in addition to offering other important immune, nutritional, and metabolic benefits (Kramer & Kakuma, 2012). Supporting exclusive breastfeeding amidst increasing urbanization and economic growth has proven challenging in neighbouring countries (Patil et al., 2015). Thus, continued support of breastfeeding practices should remain a fundamental component of future national nutrition policies and programs.

In older infants and young children (6–23 months), the number of food groups reportedly consumed in the previous 24 hr rarely met the UNICEF/WHO‐recommended minimum diversity threshold. Across South Asia, MDD is one of the least often met of the IYCF indicators (Dewey, 2016) and one most consistently positively associated with childhood nutritional status (Jones et al., 2014). In the Bhutan survey, MDD was the sole indicator that varied with household socio‐economic status, with children in households in the highest wealth fifth having nearly eight times greater odds as those in the lowest fifth of achieving a minimum dietary diversity. This suggests that despite promotion of economic equity and universal basic health care, low household socio‐economic status may still limit the diversity of foods routinely given to young children. Still, the low MDD rate even in wealthier groups suggests programmes that educate and support parents to improve the diversity of children's complementary feeding diets may benefit all socio‐economic strata throughout the country. Finally, although anthropometric status indicators suggest a country of mild to moderate nutritional risk, a high prevalence of anaemia—44% and 35% in children and nonpregnant women, respectively (Campbell et al., unpublished results, 2017)—has been observed, suggesting a more complex nutritional landscape exists in Bhutan requiring further investigation.

Strengths of this study include its national representation and use of an IYCF assessment module that adapted breastfeeding and diet questions from the most recent Multiple Indicator Cluster Survey (MICS5 Tools, 2017), facilitating classification and comparison of feeding practices according to accepted WHO/UNICEF IYCF criteria. A limitation is the small sample sizes achieved within some regions and areas that led to insufficient numbers in cells of contingency tables, with consequent imprecision in estimation, which precluded developing stratified and multivariable models. Limitations of the questionnaire data also inhibited our ability to examine certain indicators, such as intake of iron‐rich foods and supplements, which may have been revealing.

In the present analysis, we found comparatively better IYCF practices that likely contribute to what may be considered a “model” nutritional risk profile for the South Asia region. Still, there remains a need to continue promoting practices that can assure dietary adequacy for children under two. These should include a primary focus on promoting exclusive breastfeeding in the first 6 months of life and greater diversity in complementary foods in the subsequent three semesters.

## CONFLICTS OF INTEREST

The authors declare that they have no conflicts of interest.

## CONTRIBUTIONS

RKC, VA, YK, and KPW designed the current study; VA, LD, and VJ led the design and implementation of the National Nutrition Survey 2015; VA, LD, VJ, JW, SDG, and NH prepared and analysed the data for the survey report; RKC and YK conducted data analysis for this study; RKC wrote the manuscript. All authors read and approved the manuscript; KPW had primary responsibility for the final content.

## Supporting information

Table S1. Definitions of infant and young child feeding practice indicators (from (World Health Organization, 2010)) and modifications for NNS 2015

Table S2. Household, maternal and child characteristics in National Nutrition Survey Bhutan 2015 participants 0–24 mo of age providing IYCF data^a^

Table S3. Infant and young child feeding practice indicators for children 0–24 months in the National Nutrition Survey Bhutan 2015

Table S4. Household, maternal and child characteristics predictive of infant and young child feeding practice indicators in the National Nutrition Survey Bhutan 2015
