# Supplementary material for: Infant and young child feeding practices and nutritional status in Bhutan
Source: Matern Child Nutr. 2018 Nov 29;14(Suppl 4):e12762. doi: 10.1111/mcn.12762 (PMC6587771; doi:10.1111/mcn.12762)
Supplement: Supplementary file 1 — Table S1. Definitions of infant and young child feeding practice indicators (from (World Health Organization, 2010)) and modifications for NNS 2015 [file MCN-14-e12762-s003.docx]

Supplemental Table 1. Definitions of infant and young child feeding practice indicators (from (World Health Organization, 2010)) and modifications for NNS 2015

| **Indicator** | **Definition** | **Calculation** | **Modifications for NNS Bhutan 2015 Questionnaire** |
| --- | --- | --- | --- |
| Early initiation of breastfeeding | Proportion of children born in the last 24 mo who were put to the breast within one hour of birth | $\frac{Children born in the last 24 mo who were put to the breast within 1 hr of birth}{Children born in the last 24 mo}$ | Assessed as per guidelines |
| Exclusive breastfeeding under 6 months | Proportion of children 0-5 mo of age who are fed exclusively with breast milk | $\frac{Infants 0-5 mo of age who received only breast milk during the previous day}{Infants 0-5 mo of age}$ | NNS asked how many times child was breastfed during the day and at night rather than whether the child was breastfed during the prior day and night. Feeding of foods/beverages other than breastmilk was assessed as per the guidelines. |
| Predominant breastfeeding under 6 months | Proportion of children 0-5 mo of age who are predominantly breastfed | $\frac{\begin{aligned} Infants 0-5 mo of age who received breast milk as the predominant \\ source of nourishment during the previous day \end{aligned}}{Infants 0-5 mo of age}$ | As above. |
| Continued breastfeeding at 1 year | Proportion of children 12-15 mo of age who are fed breast milk | $\frac{Children 12-15 mo of age who received breast milk during the previous day}{Children 12-15 mo of age}$ | As above. |
| Continued breastfeeding at 2 years | Proportion of children 20-23 mo of age who are fed breast milk | $\frac{Children 20-23 mo of age who received breast milk during the previous day}{Children 20-23 mo of age}$ | As above. |
| Age-appropriate breastfeeding | Proportion of children 0-23 mo of age who are appropriately breastfed | $\frac{Infants 0-5 mo of age who received only breast milk during the previous day}{Infants 0-5 mo of age}$  **AND**  $\frac{\begin{aligned} Children 6-23 mo of age who received breast milk, as well as solid, \\ semi-solid or soft foods, during the previous day \end{aligned}}{Children 6-23 mo of age}$ | As above. |
| Fed prelacteal | Proportion of children born in the last 24 mo who were fed anything between birth and first breast milk | [not a UNICEF indicator] |  |
| Fed colostrum | Proportion of children born in the last 24 mo who were fed colostrum | [not a UNICEF indicator] |  |
| Introduction of solid, semi-solid and soft foods (timely CF introduction) | Proportion of infants 6-8 mo of age who receive solid, semi-solid or soft foods | $\frac{\begin{aligned} Infants 6-8 mo of age who received solid, semi-solid or soft \\ foods during the previous day \end{aligned}}{Infants 6-8 mo of age}$ | Assessed as per guidelines |
| Minimum dietary diversity | Proportion of children 6-23 mo of age who receive foods from 4 or more food groups | $\frac{\begin{aligned} Children 6-23 mo of age who received foods from \geq4 food \\ groups during the previous day \end{aligned}}{Children 6-23 mo of age}$ | Assessed as per guidelines |
| Minimum meal frequency | Proportion of children 6–23 months of age who receive solid, semi-solid, or soft foods the mini­mum number of times | $\frac{\begin{aligned} Children 6-23 mo of age who received solid, semi-solid or soft \\ foods the minium number of times or more during the previous day \end{aligned}}{Breastfed children 6-23 mo of age}$ | Meal frequency minimums: ≥2 meals for breastfed children 6-8 mo; ≥3 meals for breastfed children 9-23 mo; ≥4 meals for non-breastfed children 6-23 mo.  Milk feeding frequency was not queried in NNS as feeding animal milks is not common in Bhutan. For non-breastfed children, only frequency of solid, semi-solid and soft foods fed in the prior day were counted towards the meal frequency cutoff. |
| Minimum acceptable diet | Proportion of children 6–23 months of age who receive a minimum acceptable diet | $\frac{\begin{aligned} Children 6-23 mo of age who had at least the minimum dietary \\ diversity and the minimum meal frequency during the previous day \end{aligned}}{Breastfed children 6-23 mo of age}$ | As above. |
