# Supplementary material for: Infant and young child feeding practices and nutritional status in Bhutan
Source: Matern Child Nutr. 2018 Nov 29;14(Suppl 4):e12762. doi: 10.1111/mcn.12762 (PMC6587771; doi:10.1111/mcn.12762)
Supplement: Supplementary file 2 — Table S2. Household, maternal and child characteristics in National Nutrition Survey Bhutan 2015 participants 0–24 mo of age providing IYCF dataa [file MCN-14-e12762-s001.docx]

Supplemental Table 2. Household, maternal and child characteristics in National Nutrition Survey Bhutan 2015 participants 0-24 mo of age providing IYCF data^a^

|  | **National** | **Region** | | | **Area** | |
| --- | --- | --- | --- | --- | --- | --- |
|  |  | **West** | **Central** | **East** | **Urban** | **Rural** |
|  | **n (%)^b^** | **n (%)** | **n (%)** | **n (%)** | **n (%)** | **n (%)** |
| *Maximum n* | *441* | *133* | *102* | *206* | *83* | *358* |
| **Household** | | | | | | |
| Wall material, cement^c^ | 127 (28.8) | 56 (67.5) | 71 (19.8) | 51 (38.3) | 37 (36.3) | 39 (18.9) |
| Room count, mean (SD) | 3.0 (1.7) | 3.1 (1.5) | 3.4 (1.8) | 2.8 (1.7) | 2.6 (1.2) | 3.1 (1.8) |
| Owns land | 340 (77.1) | 88 (66.2) | 80 (78.4) | 172 (83.5) | 29 (34.9) | 311 (86.9) |
| Owns car | 73 (16.6) | 34 (25.6) | 18 (17.6) | 21 (10.2) | 34 (41.0) | 39 (10.9) |
| Owns television | 290 (65.8) | 101 (75.9) | 81 (79.4) | 108 (52.4) | 79 (95.2) | 211 (58.9) |
| Owns cattle, buffalo or yaks | 406 (92.1) | 116 (87.2) | 97 (95.1) | 193 (93.7) | 82 (98.8) | 324 (90.5) |
| Owns poultry | 347 (78.7) | 117 (88.0) | 65 (63.7) | 165 (80.1) | 81 (97.6) | 266 (74.3) |
| Wealth index quintiles^d^ |  |  |  |  |  |  |
| Poorest | 146 (33.1) | 32 (24.1) | 21 (20.6) | 93 (45.1) | 0 (0.0) | 146 (40.8) |
| Poor | 139 (31.5) | 41 (30.8) | 37 (36.3) | 61 (29.6) | 6 (7.2) | 133 (37.2) |
| Medium | 57 (12.9) | 15 (11.3) | 18 (17.6) | 24 (11.7) | 20 (24.1) | 37 (10.3) |
| Wealthy | 52 (11.8) | 16 (12.0) | 16 (15.7) | 20 (9.7) | 27 (32.5) | 25 (7.0) |
| Wealthiest | 47 (10.7) | 29 (21.8) | 10 (9.8) | 8 (3.9) | 30 (36.1) | 17 (4.7) |
| Improved sanitation | 296 (67.1) | 83 (62.4) | 66 (64.7) | 147 (71.4) | 60 (72.3) | 236 (65.9) |
| Improved water source^e^ | 370 (83.9) | 107 (80.5) | 85 (83.3) | 178 (86.4) | 67 (80.7) | 303 (84.6) |
| Food insecurity^f^ | 17 (3.9) | 0 (0.0) | 17 (4.7) | 7 (5.3) | 2 (2.0) | 8 (3.9) |
| **Maternal** | | | | | | |
| Education |  |  |  |  |  |  |
| None | 178 (40.4) | 58 (43.6) | 44 (43.1) | 76 (36.9) | 20 (24.1) | 158 (44.1) |
| Primary/informal/monastic | 143 (32.4) | 33 (24.8) | 29 (28.4) | 81 (39.3) | 15 (18.1) | 128 (35.8) |
| High school+ | 116 (26.3) | 41 (30.8) | 28 (27.5) | 47 (22.8) | 47 (56.6) | 69 (19.3) |
| ANC ≥4 visits | 359 (81.4) | 116 (87.2) | 87 (85.3) | 156 (75.7) | 74 (89.2) | 285 (79.6) |
| **Child** | | | | | | |
| Sex, female | 222 (50.3) | 64 (48.1) | 51 (50.0) | 107 (51.9) | 37 (44.6) | 185 (51.7) |
| Age |  |  |  |  |  |  |
| 0-5 mo | 94 (21.3) | 19 (22.9) | 75 (20.9) | 35 (26.3) | 15 (14.7) | 44 (21.4) |
| 6-11 mo | 117 (26.5) | 19 (22.9) | 98 (27.4) | 34 (25.6) | 33 (32.4) | 50 (24.3) |
| 12-23 mo | 230 (52.2) | 45 (54.2) | 185 (51.7) | 64 (48.1) | 54 (52.9) | 112 (54.4) |
| Stunting (LAZ < -2)^g^ | 66 (15.0) | 21 (15.8) | 6 (5.9) | 39 (18.9) | 9 (10.8) | 57 (15.9) |
| Wasting (WLZ < -2) | 24 (5.4) | 8 (6.0) | 5 (4.9) | 11 (5.3) | 3 (3.6) | 21 (5.9) |
| Underweight (WAZ < -2) | 39 (8.8) | 12 (9.0) | 7 (6.9) | 20 (9.7) | 5 (6.0) | 34 (9.5) |
| Overweight (WLZ > 2) | 25 (5.7) | 8 (6.0) | 2 (2.0) | 15 (7.3) | 4 (4.8) | 21 (5.9) |

^a^Crude estimates not adjusted for survey structure. ^b^Values are n (%) in the specified category unless otherwise noted. ^c^Referrent mud/wood/other. ^d^Household asset score generated with principal component analysis (PCA) as was done in the original analysis of the survey data (Nutrition Program, 2015) but restricted to households with a child <5 years old who had anthropometry assessed. ^e^Bhutan-specific definition: piped water into the household. ^f^Food insecurity defined as one or more affirmative answers to the 4-item food security assessment. ^g^Oulying anthropometric values (LAZ <-6 or >6, WAZ >5 or <-6, WLZ <-5 or >5) were omitted.
