# Supplementary material for: Infant and young child feeding practices and nutritional status in Bhutan
Source: Matern Child Nutr. 2018 Nov 29;14(Suppl 4):e12762. doi: 10.1111/mcn.12762 (PMC6587771; doi:10.1111/mcn.12762)
Supplement: Supplementary file 3 — Table S3. Infant and young child feeding practice indicators for children 0–24 months in the National Nutrition Survey Bhutan 2015 [file MCN-14-e12762-s002.docx]

Supplemental Table 3. Infant and young child feeding practice indicators for children 0-24 months in the National Nutrition Survey Bhutan 2015

| **Indicator^a^** | **Inclusive ages (mo)** | **National** | **Region** | | | **Area** | | **Sex** | |
| --- | --- | --- | --- | --- | --- | --- | --- | --- | --- |
|  |  |  | **West** | **Central** | **East** | **Urban** | **Rural** | **Male** | **Female** |
|  |  | **Proportion (SE)^b^** | **Proportion (SE)** | **Proportion (SE)** | **Proportion (SE)** | **Proportion (SE)** | **Proportion (SE)** | **Proportion (SE)** | **Proportion (SE)** |
| *Maximum n* |  | *441* | *133* | *102* | *206* | *83* | *358* | *219* | *222* |
| Early initiation of BF | 0-23 | 0.78 (0.03) | 0.78 (0.05) | 0.78 (0.08) | 0.78 (0.03) | 0.81 (0.03) | 0.75 (0.03) | 0.77 (0.03) | 0.78 (0.05) |
| Ever breastfed | 0-23 | 0.99 (0.00) | 0.99 (0.01) | 1.00 (0.00) | 0.99 (0.00) | 1.00 (0.00) | 0.99 (0.01) | 1.00 (0.00) | 0.99 (0.01) |
| Exclusive BF < 6 mo | 0-5 | 0.52 (0.14) | 0.36 (0.19) | 0.49 (0.08) | 0.77 (0.05) | 0.53 (0.20) | 0.51 (0.07) | 0.68 (0.15) | 0.33 (0.14) |
| Predominant BF < 6 mo | 0-5 | 0.66 (0.14) | 0.47 (0.18) | 0.64 (0.12) | 0.92 (0.04) | 0.66 (0.21) | 0.65 (0.08) | 0.78 (0.12) | 0.49 (0.19) |
| Continued BF, 1 yr. | 12-15 | 0.92 (0.03) | 0.92 (0.06) | 0.94 (0.07) | 0.91 (0.03) | 0.95 (0.05) | 0.89 (0.04) | 1.00 (0.00) | 0.87 (0.05) |
| Continued BF, 2 yr. | 20-23 | 0.61 (0.07) | 0.60 (0.13) | 0.70 (0.05) | 0.56 (0.09) | 0.50 (0.13) | 0.69 (0.07) | 0.68 (0.09) | 0.52 (0.16) |
| Age-appropriate BF | 0-23 | 0.71 (0.06) | 0.66 (0.12) | 0.71 (0.03) | 0.77 (0.03) | 0.69 (0.09) | 0.73 (0.03) | 0.77 (0.04) | 0.64 (0.09) |
| Fed prelacteal | 0-23 | 0.04 (0.01) | 0.04 (0.01) | 0.02 (0.02) | 0.04 (0.01) | 0.02 (0.01) | 0.06 (0.02) | 0.02 (0.01) | 0.05 (0.02) |
| Fed colostrum | 0-23 | 0.95 (0.01) | 0.96 (0.01) | 0.87 (0.02) | 0.99 (0.00) | 0.97 (0.01) | 0.94 (0.02) | 0.96 (0.02) | 0.95 (0.02) |
| Timely introduction of CF | 6-8 | 0.93 (0.04) | 0.94 (0.07) | 0.86 (0.08) | 1.00 (0.00) | 0.97 (0.04) | 0.91 (0.06) | 0.89 (0.09) | 0.98 (0.02) |
| Minimum dietary diversity | 6-23 | 0.18 (0.04) | 0.22 (0.07) | 0.08 (0.05) | 0.18 (0.04) | 0.24 (0.04) | 0.13 (0.03) | 0.24 (0.07) | 0.11 (0.02) |
| Minimum meal frequency | 6-23 | 0.75 (0.03) | 0.76 (0.05) | 0.75 (0.04) | 0.75 (0.04) | 0.67 (0.05) | 0.82 (0.02) | 0.80 (0.03) | 0.70 (0.04) |
| Minimum acceptable diet | 6-23 | 0.16 (0.03) | 0.20 (0.06) | 0.05 (0.03) | 0.18 (0.04) | 0.21 (0.04) | 0.12 (0.03) | 0.23 (0.06) | 0.09 (0.02) |

^a^Full indicator definitions can be found in Supplemental Table 1. ^b^Proportions and standard errors generated with the “svy: proportion …” command for survey-adjusted estimates. Abbreviations: BF, breastfeeding; CF, complementary feeding.
