# Supplementary material for: Infant and young child feeding practices and nutritional status in Bhutan
Source: Matern Child Nutr. 2018 Nov 29;14(Suppl 4):e12762. doi: 10.1111/mcn.12762 (PMC6587771; doi:10.1111/mcn.12762)
Supplement: Supplementary file 4 — Table S4. Household, maternal and child characteristics predictive of infant and young child feeding practice indicators in the National Nutrition Survey Bhutan 2015 [file MCN-14-e12762-s004.docx]

Supplemental Table 4. Household, maternal and child characteristics predictive of infant and young child feeding practice indicators in the National Nutrition Survey Bhutan 2015

| **Characteristics** | **Exclusive breastfeeding < 6 mo** | **Continued breastfeeding at 2 yr** | **Minimum dietary diversity** | **Minimum meal frequency** |
| --- | --- | --- | --- | --- |
|  | **OR (95% CI)^a^** | **OR (95% CI)** | **OR (95% CI)** | **OR (95% CI)** |
| Inclusive ages (mo) | 0-5 | 20-23 | 6-23 | 6-23 |
| *Maximum n* | *94* | *73* | *347* | 325 |
| Region |  |  |  |  |
| West | 1.0 | 1.0 | 1.0 | 1.0 |
| Central | 1.7 (0.1, 27.8) | 1.6 (0.2, 10.3) | 0.3 (0.0, 3.7) | 0.9 (0.3, 2.7) |
| East | 5.9 (0.4, 95.2) | 0.8 (0.1, 6.3) | 0.8 (0.2, 3.8) | 1.0 (0.3, 2.8) |
| p-value | 0.226 | 0.560 | 0.575 | 0.990 |
| Area |  |  |  |  |
| Urban | 1.0 | 1.0 | 1.0 | 1.0 |
| Rural | 0.9 (0.1, 8.1) | 2.3 (0.2, 24.4) | 0.5 (0.2, 1.3) | 2.2 (0.7, 6.8) |
| p-value | 0.919 | 0.346 | 0.094 | 0.102 |
| Wealth index |  |  |  |  |
| Lowest | 1.0 | 1.0 | 1.0 | 1.0 |
| Low | 0.8 (0.0, 19.1) | 0.1 (0.0, 17.3) | 4.2 (0.4, 45.1) | 1.1 (0.1, 9.4) |
| Medium | 3.7 (0.2, 83.9) | 0.1 (0.0, 8.2) | 2.0 (0.2, 16.5) | 0.4 (0.1, 2.5) |
| High | 0.5 (0.0, 11.8) | 0.2 (0.0, 3.6) | 5.3 (1.1, 25.2) | 0.4 (0.1, 1.0) |
| Highest | 1.0 (0.0, 25.3) | 0.3 (0.0, 12.8) | 7.8 (1.3, 46.9) | 0.8 (0.3, 2.5) |
| p-value^b^ |  |  |  |  |
| Number of rooms |  |  |  |  |
| ≤3 | 1.0 | 1.0 | 1.0 | 1.0 |
| >3 | 0.4 (0.0, 4.1) | 1.6 (0.2, 12.5) | 1.2 (0.2, 8.3) | 1.3 (0.3, 6.0) |
| p-value | 0.314 | 0.504 | 0.828 | 0.632 |
| Improved sanitation |  |  |  |  |
| No | 1.0 | 1.0 | 1.0 | 1.0 |
| Yes | 1.2 (0.1, 10.5) | 0.5 (0.1, 3.4) | 2.6 (0.7, 9.4) | 1.4 (0.3, 7.2) |
| p-value | 0.764 | 0.315 | 0.097 | 0.526 |
| Improved water^c^ |  |  |  |  |
| No | 1.0 | 1.0 | 1.0 | 1.0 |
| Yes | 2.0 (0.2, 17.3) | 0.5 (0.1, 2.5) | 1.5 (0.3, 7.9) | 1.2 (0.3, 4.0) |
| p-value | 0.382 | 0.270 | 0.522 | 0.698 |
| Food insecurity |  |  |  |  |
| No | 1.0 | 1.0 | 1.0 | 1.0 |
| Yes | 0.0 (0.0, 11.8) | 0.2 (0.0, 2.6) | 1.1 (0.0, 73.0) | 0.5 (0.0, 15.7) |
| p-value | 0.160 | 0.137 | 0.973 | 0.601 |
| Mother's education |  |  |  |  |
| None | 1.0 | 1.0 | 1.0 | 1.0 |
| Primary^d^ | 1.1 (0.2, 7.2) | 0.1 (0.0, 0.8) | 2.3 (0.2, 22.8) | 0.8 (0.1, 6.0) |
| High School+ | 1.3 (0.2, 7.8) | 0.2 (0.0, 1.9) | 2.5 (0.7, 8.5) | 0.9 (0.2, 4.7) |
| p-value | 0.909 | 0.182 | 0.327 | 0.963 |
| Antenatal care |  |  |  |  |
| <4 visits | 1.0 | 1.0 | 1.0 | 1.0 |
| ≥4 visits | 0.3 (0.0, 4.5) | 0.1 (0.0, 7.6) | 0.9 (0.0, 17.7) | 0.5 (0.1, 2.6) |
| p-value | 0.269 | 0.186 | 0.906 | 0.292 |
| Child age (<6 mo) |  |  |  |  |
| 0-3 mo | 1.0 | - | - | - |
| 4-5 mo | 0.6 (0.1, 4.1) | - | - | - |
| p-value | 0.439 |  |  |  |
| Child age (6-23 mo) |  |  |  |  |
| 6-11 mo | - | - | 1.0 | 1.0 |
| 12-23 mo | - | - | 1.6 (0.6, 5.7) | 1.5 (0.4, 4.8) |
| p-value |  |  | 0.312 | 0.386 |
| Child sex |  |  |  |  |
| Male | 1.0 | 1.0 | 1.0 | 1.0 |
| Female | 0.2 (0.1, 0.9) | 0.5 (0.0, 6.7) | 0.4 (0.1, 1.5) | 0.6 (0.2, 1.3) |
| p-value | 0.044 | 0.475 | 0.108 | 0.131 |

^a^Models are logistic regressions adjusted for the survey design using the “svy:” prefix and developed separately for each household characteristic. ^b^Models for socioeconomic status quintiles had insufficient degrees of freedom to calculate p-values. ^c^Bhutan-specific definition: piped water into the household. ^d^Primary schooling includes government primary schooling and informal or monastic education.
